# Facile Fabrication of Flexible Electrodes and Immobilization of Silver Nanoparticles on Nanoscale Silicate Platelets to Form Highly Conductive Nanohybrid Films for Wearable Electronic Devices

**DOI:** 10.3390/nano10010065

**Published:** 2019-12-27

**Authors:** Peng-Yang Huang, Chih-Wei Chiu, Chen-Yang Huang, Sheng-Yen Shen, Yen-Chen Lee, Chih-Chia Cheng, Ru-Jong Jeng, Jiang-Jen Lin

**Affiliations:** 1 Institute of Polymer Science and Engineering, National Taiwan University, Taipei 10617, Taiwan; d02549006@ntu.edu.tw (P.-Y.H.); reggieshen7@gmail.com (S.-Y.S.); 2 Department of Materials Science and Engineering, National Taiwan University of Science and Technology, Taipei 10607, Taiwan; d10504015@gapps.ntust.edu.tw (C.-Y.H.); d10404013@mail.ntust.edu.tw (Y.-C.L.); 3 Graduate Institute of Applied Science and Technology, National Taiwan University of Science and Technology, Taipei 10607, Taiwan; cccheng@mail.ntust.edu.tw; 4 Department of Materials Science and Engineering, National Chung Hsing University, Taichung 40227, Taiwan

**Keywords:** silicate nanoplatelets, silver nanoparticles, dispersion, nanohybrid film, electrical conductivity, electrocardiogram

## Abstract

This study investigated films with remarkably high electrical conductivity after they were easily prepared from organic/inorganic nanohybrid solutions containing an organic polymeric dispersant and two-dimensional nanoscale silicate platelets as the inorganic stabilizer dispersed with silver nanoparticles. Transmission electron microscopy shows that the production of silver nanoparticles synthesized by the in situ chemical reduction of AgNO_3_ in an aqueous solution by *N,N*-dimethylformamide results in an average silver nanoparticle diameter of circa 20 nm. Thin films of silver nanoparticles were prepared on a 1-μm-thick film with a low sheet resistance of 8.24 × 10^−4^ Ω/sq, achieved through the surface migration of silver nanoparticles and prepared by sintering at 300 °C to form an interconnected network. This was achieved with a silver nanoparticle content of 5 wt%, using nanoscale silicate platelets/polyoxyethylene-segmented polyimide/AgNO_3_ at a weight ratio of 1:10:35. During sintering, the color of the hybrid film changed from gold to milky white, suggesting the migration of silver nanoparticles and the formation of an interconnected network. The results show promise for the fabrication of novel silver-based electrocardiogram electrodes and a flexible wireless electrocardiogram measurement system for wearable electronics.

## 1. Introduction

Wearable electronic devices, capable of monitoring motion, heart rate, blood pressure, wrist pulse, and other health-related parameters are an integral part of healthcare [1,2]. Cardiovascular disease is among the leading causes of death worldwide and accounts for more than half of all non-communicable disease fatalities [3]. In order to provide treatment, patients are often monitored using medical electrocardiography (ECG) [4]. The ECG waveform shows the electrical activity of the heart and can detect heart irregularities. In order to fabricate sensor devices that transmit physiological signals, studies have explored the use of silver fibers [5], stainless steel [6], intrinsically conductive polymers [7], carbon-based nanomaterials [8], and other nanomaterials for use as electrodes [9,10]. These devices, based on the immobilization of silver nanoparticles, can sense physiological signals in the human body [11]. Reducing organic substrate-based compounds and their use in fabricating nanostructured materials is well-documented for the synthesis of Cu [12,13], Fe [14,15], and Ag [16,17] nanoparticles. In response to the growing interest in wearable electronic devices, and to assure the dispersion of nanoscale particles in solution, a suitable stabilizing agent is needed [18]. Bulk silver materials are commonly utilized in the microelectronics industry due to their low sheet resistance [19]. However, it is arduous to prepare Ag-coated substrate films with an electrical resistance as low as 10^−2^ Ω/sq [20] due to their unstable electrical characteristics. Therefore, temperatures higher than 300 °C are necessary during operation of power electronics coated with Ag films; providing scope for improvement in performance and viability of Ag films through the development of new Ag-coated functional materials [21,22].

Synthesizing silver nanoparticles (AgNPs) using inorganic stabilizing agents such as Cu [23], Sn [24], carbon nanotubes [25], Al_2_O_3_ [26], and layered silicate clay [27,28] are approaches that avoid the use of organic components. Achieving a low-temperature melting point of the particles was a promising development, made possible due to the sintering properties of AgNPs on the surface of clay platelets [29]. Exfoliated silicate platelets with active plate-chips can exhibit highly charged ions suitable for attracting with AgNPs. The direct exfoliation process of layered Na^+^-MMT by a polyamine salt ion-exchange reaction [30] Pinvolves the isolation of clay nanoplatelets and results in a material with a width of 100 nm and a thickness of 1 nm [31]. These platelet-like nanosheets, which possess surface functionalities due to surface charges constructed of ≡SiO^−^Na^+^, have a high tendency for polar organic and inorganic salts including AgNO_3_ [16,29]. However, it is facile to achieve sheet resistance for transmittance with films, because the low Ag content in these sheets cannot form an interlinked network of AgNPs.

Herein, the manufacture of well-dispersed nanohybrid solutions of AgNPs based on inorganic silicate nanoplatelets and organic polymeric surfactants as hybrid dispersants is studied. The organic surfactant, a polyoxyethylene-segmented polyimide (POE-imide), was prepared from an 4,4’-oxydiphthalic anhydride (ODPA) and polyoxyethylene diamine (POE-amine) by an imidation reaction. The hybrid dispersants of polymeric surfactant and silicate nanoplatelet result in a well- dispersed solution of AgNPs. Furthermore, the hybrid dispersion was used to form films, and the melting properties were explored by field-emission scanning electron microscopy. The low-resistance sheet films were successfully fabricated due to Ag melting, thereby enhancing the formation of a continuous Ag interlinked network structure. Thus, a novel silver-based film was fabricated for use in a flexible ECG electrode and a wireless ECG analysis system for wearable electronic devices.

## 2. Materials and Methods

### 2.1. Reagents and Chemicals

Sodium montmorillonite (Na^+^-MMT; Nanocor Co., Chicago, IL, USA) is a Na^+^ type of layered aluminosilicate clay with a cationic exchange capacity (CEC) of 115 meq/100 g. The 2:1 type of layered silicate clay comprises two tetrahedron sheets sandwiching an edge-shared octahedral sheet, with an average polydisperse dimension of 100 nm × 100 nm × 1 nm for each sheet and approximately 10 platelets in one stack. The 4,4’-Oxydiphthalic anhydride (ODPA) was purchased from Aldrich Chemical Co., St. Louis, MO, USA, and purified by sublimation. Jeffamine^®^ ED2001 (abbreviated POE-2000), a commercial brand of POE-amine, was purchased from Huntsman Chemical Co., Los Angeles, CA, USA. Silver nitrate (AgNO_3_, purity 99.9%) was purchased from Aldrich Chemical Co., St. Louis, MO, USA, and *N,N*-dimethylformamide (DMF) from TEDIA Company Inc., Fairfield, OH, USA.

### 2.2. Direct Exfoliation of Layered Na^+^-MMT Clay into Nanoscale Silicate Platelets in Water Suspension

Previously, characterization of the direct exfoliation of synthetic Na^+^-MMT to obtain exfoliated nanoscale silicate platelets (NSPs) using a polyamine salt has been demonstrated [30,31,32]. NSPs were dispersed in water and purified by solvent toluene/water extraction. Subsequently, the surface ionic character of the NSPs was determined using a zeta potential analyzer (Zetasizer Nano-Zs90 instrument, Malvern Instrument Ltd., Worcestershire, UK). X-ray powder diffraction (XRD), transmission electron microscopy (TEM), and atomic force microscopy (AFM) were used to investigate which materials could be exfoliated. The ionic NSPs had a measured width of approximately 100 nm and thickness of 1 nm.

### 2.3. Synthesis of the Poly(oxyethelene)-Segmented Amide–Imide

**Scheme 1:**
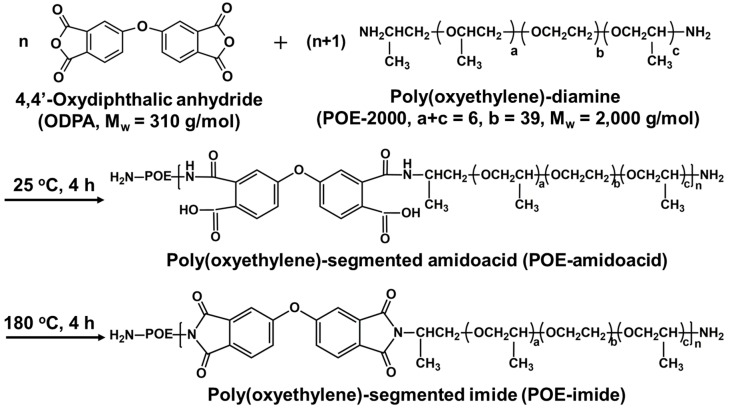
Synthetic scheme for converting 4,4’-oxydiphthalic anhydride and poly(oxyethylene)-diamine to the poly(oxyethylene alkylene)-segmented amido acid and imide (polyoxyethylene-(POE)-derived amido acid and imide) as polymeric dispersants.

**Figure 1:**
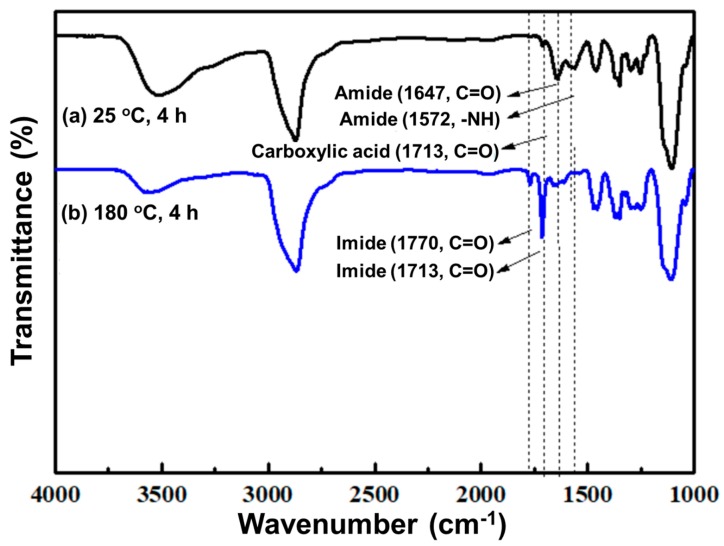
Fourier transform infrared (FTIR) spectra of POE-derived dispersants of (**a**) POE-amido acid intermediate and (**b**) POE-imide from amido acid to cyclized imide functionalities.

A poly(oxyethelene)-segmented amide–imide was synthesized by the amidation and following imidation of ODPA and POE-2000 in a molar ratio of 5:6 (Scheme 1). For this, 10 g of 0.005 mol POE-2000 in 15 mL tetrahydrofuran was transferred to a 150 mL three-neck round-bottom flask equipped with a thermometer, a mechanical stirrer, and nitrogen outlet/inlet line. Next, 1.29 g of 0.004 mol solution of ODPA dispersed in 12 mL tetrahydrofuran was added dropwise to the flask through an addition pipe with vigorous stirring and maintained at a temperature of 25 °C for 4 h. The product of the reaction was analyzed via Fourier transform infrared (FTIR) spectroscopy, showing characteristic absorption peaks at 1572 cm^−1^ and 1647 cm^−1^ for the amido acid functionalities (Figure 1). Major absorption peaks were detected for cyclized imide functionalities at 1713 cm^−1^ and 1770 cm^−1^, after increasing temperature to 180 °C for 4 h. The peaks, which indicated a significant conversion of POE-imide, had different characteristics. A yellow solid was recovered after the product solution underwent rotary evaporation.

### 2.4. Preparation of NSP/POE-Imide/AgNP Nanohybrid Suspensions

The NSP slurry (0.025 wt% of NSP) was dispersed in 5 g of water in a 50 mL volumetric flask and left at an ambient temperature for 10 min; similarly, 0.25 g of POE-imide was dispersed in 5 g of DMF. Next, the NSP and POE-imide solutions were mixed, followed by the addition of AgNO_3_ (1.49 g, 5 wt%). The temperature was maintained at 80 °C for 4 h and stirred. Color changes from yellow-brown to dark golden were visually assessed as a function of temperature; the suspension was analyzed using a UV–Vis spectrophotometer, which suggested the reduction of Ag^+^ to Ag^0^ when the color changed from white to yellow. A homogeneous suspension was observed in solutions of AgNO_3_, POE-imide, and NSP, as well as sintering processes that were unique to nanoparticles. In addition, a control experiment using POE-imide stabilized homogenous colloidal AgNP solution was conducted. The UV absorption spectra of the various suspensions were observed using 0.2 g of the samples in 5.0 g of distilled water. Transmission electron microscopy (TEM) images were acquired for further analysis.

### 2.5. Preparation of NSP/POE-Imide/AgNP Nanohybrid Films

The film was attached to a polyimide substrate of area 2 × 2 cm. The dripping solution of the nanohybrid solution was attached on a piece of the substrate via high-temperature sintering and then placed in a convection oven. The sintering programs were set to 80 °C, 160 °C, 250 °C, and 300 °C for 0.5 h each prior to use.

### 2.6. Instrumental Characterization

Fourier transform infrared spectroscopy (FTIR) was carried out on a Perkin–Elmer Spectrum One FTIR Spectrometer (PerkinElmer Inc., Waltham, MA, USA) between 4000–400 cm^−1^. The POE-imide dispersants were prepared by dissolving in ethanol, followed by evaporation on a KBr plate resulting in a thin film. The UV–Vis spectra of the AgNP suspension was measured using a Shimadzu UV-2450 spectrophotometer (Shimadzu Co. Ltd., Tokyo, Japan). TEM images were observed using a Zeiss EM 902A field emission microscope (Carl Zeiss Microscopy GmbH, Oberkochen, Germany) at an accelerating voltage of 80 kV. Field emission scanning electron microscopy (FE-SEM) images were observed using a JEOL JSM-6700F scanning electron microscope (JEOL, Tokyo, Japan) operating at an accelerating voltage of 80 kV. The sample substrates were mounted on a sample holder using adhesive carbon tape. Elemental analyses of the film measurements were conducted using energy-dispersive X-ray spectroscopy (EDX, Oxford INCA Energy 400, Oxford Instrument plc, Abingdon, UK). Thermogravimetric analysis (TGA), was conducted on a Perkin–Elmer Pyris 1 instrument. The organic/inorganic weight ratio was estimated from the weight losses by ramping the temperature from 100 to 500 °C at the rate of 20 °C/min in nitrogen. The sheet resistances of the hybrid films containing AgNPs were surveyed with a Keithley 2400 digital source meter (Keithley Instruments, Inc., Cleveland, OH, USA) equipped with a four-point probe. The electrical conductivities of the hybrid films containing AgNPs was measured by utilizing them to illuminate light-emitting diode (LED) lamps with power dissipation at 100 mW and a peak forward current of 100 mA. Triplicate measurements of the synthesized quantities were performed to confirm reproducibility.

## 3. Results and Discussion

### 3.1. Synthesis of NSP/POE-Imide/AgNP Nanohybrid Suspensions

**Scheme 2:**
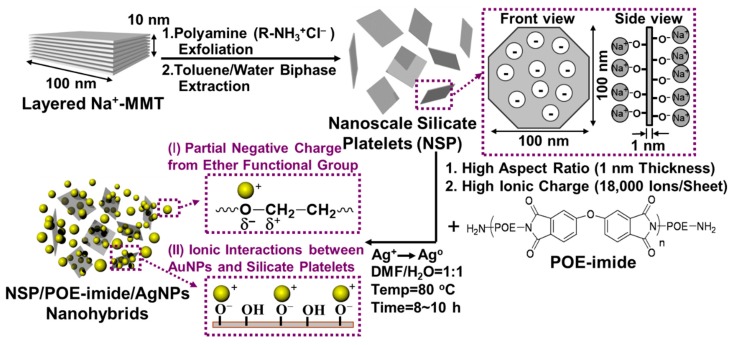
Schematic of the exfoliation process for the layered Na^+^-MMT clay into exfoliated silicate nanosheets and illustration of the surface ionic charges. The polymeric dispersant and silicate are attracted to Ag^+^ ions, resulting in well-dispersed AgNPs. A schematic of the dispersion mechanisms of the AgNPs/nanoscale silicate platelets (NSPs) and AgNPs/POE-imide stabilizations are also shown.

Nanoscale silicate platelets were fabricated using a two-step process, including the exfoliated agent of layered Na^+^-MMT, followed by organic solvent extraction using a water and toluene mixture (Scheme 2) [30,31,32]. During extraction, the mixture separated into aqueous and organic phases. The upper layer, containing the exfoliated agent organics was evaporated, leaving the lower aqueous layer, which contained the NSP suspension. The nanoplates consisted of 18,000 ions/plate and exhibited ionic interactions arising from the in situ reduction of silver nitrate used in the synthesis of AgNPs. The AgNPs were placed onto both the NSP and the polymeric dispersant because of their superior non-covalent ionic interactions and van der Waals force. Species possessing mutual attraction through non-covalent interactions were needed so that the two nanomaterials successfully engineered a substrate for the electrostatic attraction between the AgNPs and NSPs. Additionally, there is a partial negative charge in the suspension from the ether functional group of the POE (–CH_2_CH_2_O–)R_x_R block fabricated on POE-imide/AgNPs.

**Figure 2:**
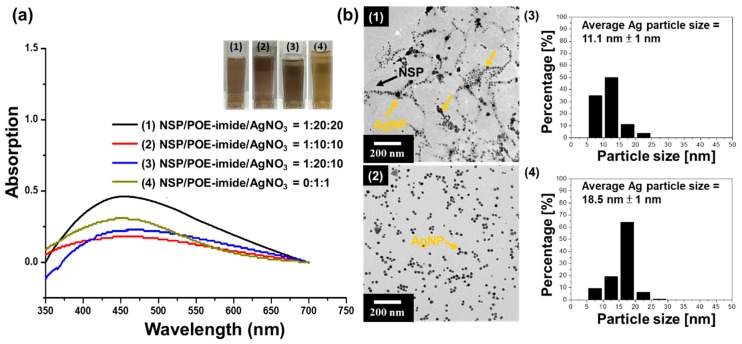
(**a**) UV–Vis absorption spectra of AgNPs suspension with various weight fractions of POE-imide and NSP. Inset: NSP/POE-imide/AgNO_3_ reductions with weight ratios of (1) 1:20:20, (2) 1:10:10, (3) 1:20:10, and (4) 0:1:1. (**b**) TEM images of NSP/POE-imide/AgNO_3_ with weight ratios of (1) 1:20:20 and (2) 0:1:1. Histograms of the Ag particle sizes, determined from TEM images of NSP/POE-imide/AgNO_3_ with weight ratios of (3) 1:20:20 and (4) 0:1:1.

Key to this synthesis is the binding of Ag^+^ ions to the ionic NSP surface and the polyoxyethylene backbone amine, which proceeds through the chemical reduction of DMF and simultaneous reduction of Ag^+^ to Ag^0^. DMF can be used as a reducing agent for converting Ag^+^ to Ag^0^ for the in situ chemical synthesis of AgNPs when the dispersing POE-imide/NSP are sealed in a H_2_O/DMF co-solvent [16,21]. Samples with varied weight ratios were analyzed, and the color change was detected with UV–Vis spectroscopy (Figure 2a). NSP/POE-imide/AgNO_3_ was prepared in weight ratios of 1:10:10, 1:20:20, 1:20:10, and 0:1:1. A characteristic absorption peak at 440 nm for the NSP/POE-imide/AgNO_3_ dispersion indicated a homogeneous mixture. In the control experiment, a POE-imide surfactant was utilized as the stabilizer instead of NSP/POE-imide. The product solution exhibited an absorption peak at approximately 446 nm when the POE-imide/AgNO_3_ weight ratio was maintained at 1:1. Figure 2b shows TEM images showing the size distribution of formed AgNPs. The AgNPs are well-dispersed on the surface of the platelets with a particle diameter of approximately 10–20 nm. During the reduction of AgNO_3_, the surface of the silicate sheets and the polymer chains adhere together when the AgNPs were generated and decreased simultaneously in size. The average size of the AgNPs decreased due to the increasing number of NSPs added to the system. The hybrid dispersants showed excellent homogeneity and remained stable for three months.

### 3.2. Facile Fabrication of the Highly Conductive NSP/POE-Imide/AgNPs Nanohybrid Films

**Figure 3:**
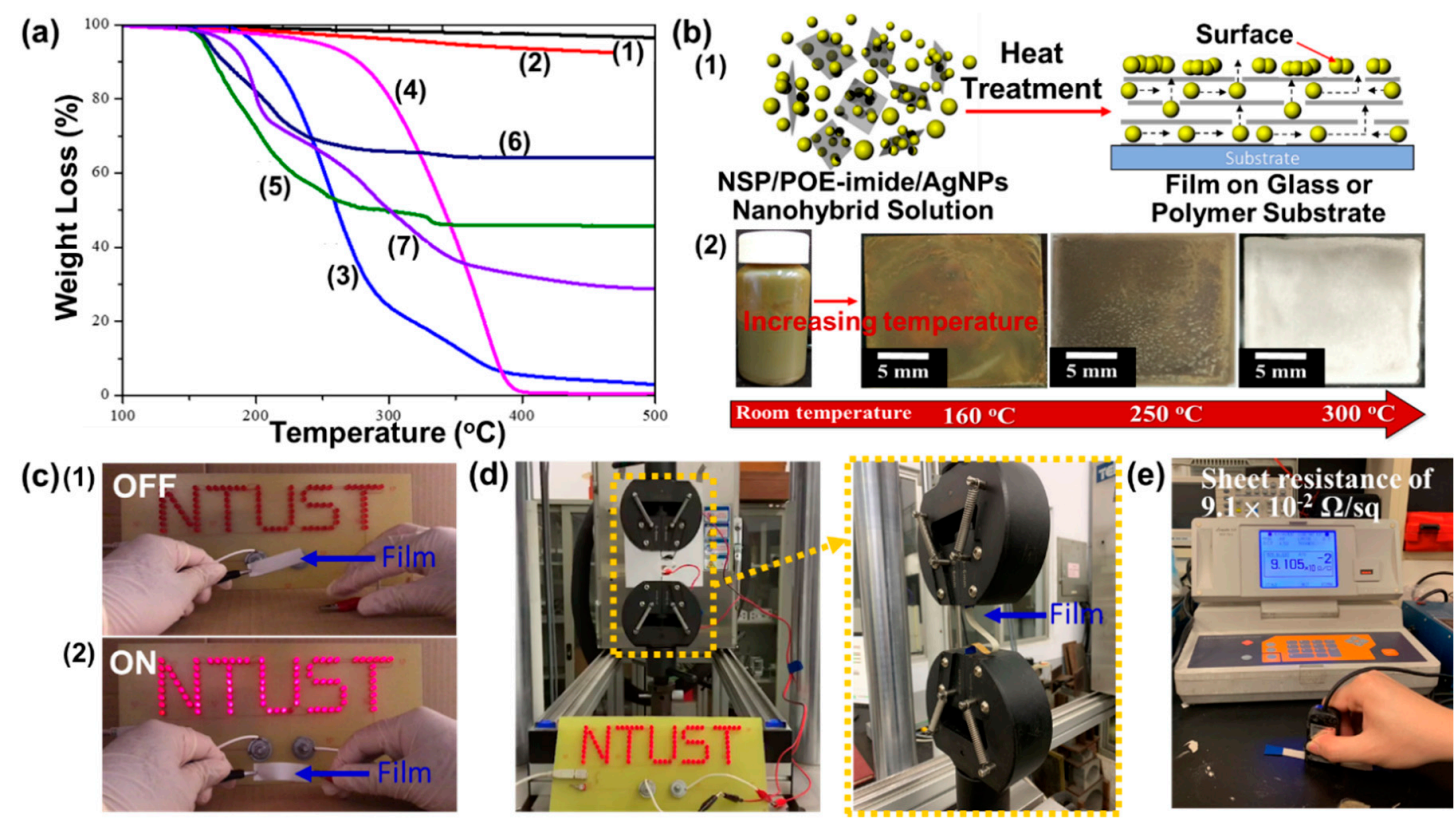
(**a**) Thermogravimetric analysis (TGA) curves and relative thermo-oxidative stability of the NSP/POE-imide/AgNPs nanohybrid films with different weight ratios in air. (1) Na^+^-MMT (non-exfoliation), (2) NSP (exfoliation), (3) POE-imide (organic dispersant), (4) POE-imide nanohybrid with NSP (exfoliation), (5) POE-imide nanohybrid with AgNP/NSP (exfoliation) in 5 wt%, (6) POE-imide nanohybrid with AgNP/NSP (exfoliation) in 8 wt%, and (7) AgNP/POE-imide. (**b**) Colored films were observed (1) schematic diagram showing the migration of AgNPs to the film surface, (2) demonstration of AgNP migration to surface and its melting to achieve white color while heating NSP/POE-imide/AgNPs. (**c**) (1 and 2) Demonstration of electrode behavior by switching LED bulbs OFF and ON of NSP/POE-imide/AgNPs films with weight ratio of 1:20:20, which have been heated to 300 °C. (**d**) The mechanical stability of flexible electrodes was tested for a flexible duration under 3000 cycles. (**e**) The result showed that the sheet resistance of hybrid films is maintained by at least 10^−2^ Ω/sq after a durability test.

Thermogravimetric analysis (TGA) was conducted in the temperature range 100–500 °C at a heating rate of 20 °C/min in nitrogen (Figure 3a). The TGA trace indicated thermal degradation at sintering temperatures of 180 °C, 200 °C, and 250 °C. It also indicated the weight ratios of the non-exfoliated and exfoliated clay control samples. NSP/POE-imide/AgNPs hybrid samples with weight ratios of 1:0:0, 0:1:0, 0:1:1, and 1:10:35 (5 wt%) were prepared through sintering treatments at different temperatures. Furthermore, the dispersion of the AgNPs/POE-imide/NSP hybrid material was coated on a polyimide substrate and subjected to a succession of heat treatments (80 °C, 160 °C, 200 °C, and 300 °C for 0.5 h each). The appearance of the film product indicated that the AgNPs had moved to the surface of the NSP and had agglomerated, forming an interconnected network structure. This was accompanied by a change in film surface color—from gold to milky white (Figure 3b (1) and (2)). The role of silicate platelets is to generate paths for directing the migration of AgNPs into the surface. However, some AgNPs attached to the silicate surface at 300 °C, as evidenced by the high conductivity. The electrical resistance of the sintered films was measured by using the films in an electrical circuit to illuminate LED bulbs (Figure 3c (1) and (2)) and Supplementary Material Video S1). Furthermore, the flexible duration and simultaneous conductance changes were tested under 3000 cycles (Figure 3d,e, and Supplementary Material Video S1). The electrical performance of the films showed a different trend for the POE-imide contents than that observed for the resistivity of the silver features. After heat treatment at 300 °C, the sheet resistance of the NSP/POE-imide/AgNO_3_ hybrid film with a weight ratio of 1:20:20 varied by over eight orders of magnitude compared to that of the hybrid film with a weight ratio of 1:0:0 (the respective sheet resistances were 1.94 × 10^6^ Ω/sq and 5.32 × 10^−2^ Ω/sq), suggesting that the sheet resistance varied according to the AgNP content. A decrease of the POE-imide content (NSP/POE-imide/AgNO_3_ 1:10:20) led to a sheet resistance of 5.56 × 10^−2^ Ω/sq when the sintering temperature was 300 °C. The sheet resistance decreased to 10^−4^ Ω/sq when the AgNP content increased to 5 wt% (NSP/POE-imide/AgNO_3_ 1:10:35) for the same temperature (Table 1). The variation in the sheet resistance shows that the conductivity of the nanohybrid materials was improved when AgNPs were used as conductive fillers.

### 3.3. Observation of Interconnected Network of Melted AgNPs via FE-SEM

**Figure 4:**
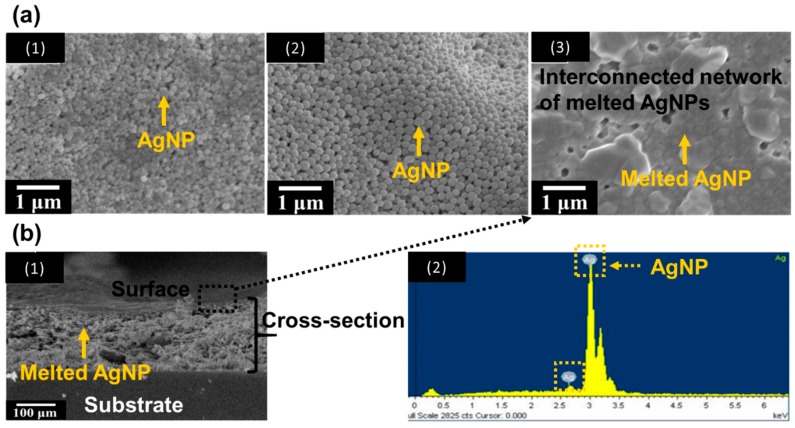
(**a**) Field emission scanning electron microscopy (FE-SEM) images of NSP/POE-imide/AgNP nanohybrid films with a weight ratio of 1:10:35 with 5 wt% AgNPs in solution and heat-treated at (**1**) 160 °C, (**2**) 250 °C, and (**3**) 300 °C for 0.5 h each. (**b**) (**1**) Cross-sectional view of nanohybrid film. (**2**) EDS profiles of the NSP/POE-imide/AgNP nanohybrid films with a weight ratio of 1:10:35 after heating at 300 °C.

Field emission scanning electron microscopy images of nanohybrid films with sintering temperatures of 160 °C, 250 °C, and 300 °C were obtained (Figure 4a). A homogeneous size distribution and a low surface energy was observed on the NSP sheets of AgNPs. The AgNPs can be seen moving to the surface of the NSP and aggregating into lumps as the temperature increased from 160 °C to 300 °C. A continuous network was observed when the AgNPs melted at 300 °C; this contributed to enhancing the conductivity of the nanohybrid materials. Furthermore, the cross-section of the hybrid films shows a layer-by-layer structure of silicate platelets, suggesting the migration of AgNPs to the surface when the temperature reached 300 °C (Figure 4b (1)). Energy dispersive spectroscopy (EDS) analysis of the hybrid films confirmed that they consisted of Ag and NSP (Figure 4b (2)). Degradation of the organic dispersant appeared to be completed at a temperature of 300 °C; furthermore, it can be shown that the interconnected network of AgNPs is formed due to the sintering temperature. Thus, the AgNPs were melted by selecting hybrid films of appropriate conductivity.

### 3.4. Measurements of ECG Signals

**Figure 5:**
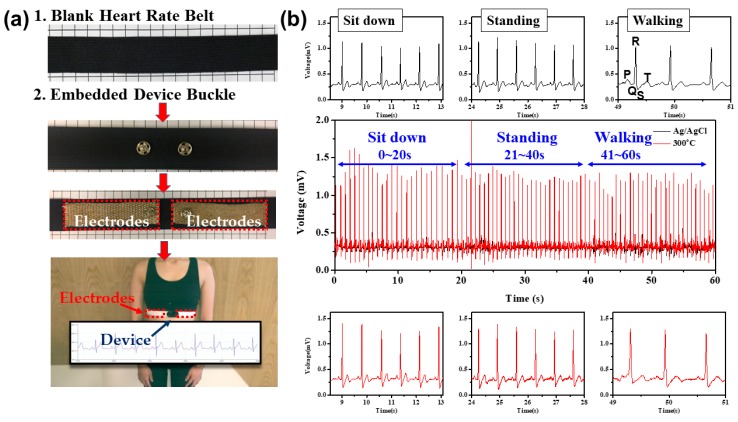
(**a**) Optical images of heart rate monitor device featuring silver-based electrodes. (**b**) Magnified view of the measured electrocardiogram (ECG) wave, showing the P-wave, QRS-complex, and T-wave under different conditions.

Electrocardiogram signals were measured from fabricated silver-based electrodes via an ECG acquisition system (ECG TD3 Device, Yang Yin Co. Ltd., Taipei, Taiwan) using manufactured smart clothing to detect measurements from two electrodes [33,34]. ECG readings were obtained from a 25-year-old female during pre-exercise, exercise, and post-exercise phases with the electrodes attached to clothing on the upper-body. ECG signals were filtered between frequencies of 0.5 and 500 Hz during sample acquisition and amplification by the signal acquisition system. AgNPs were coated onto fiberglass after immersion for 0.5 h with the following sintering temperature program: 80 °C for 1 h; 150 °C for 0.5 h; 250 °C for 0.5 h; 300 °C for 0.5 h. The P-wave, QRS-complex, and T-wave were clearly visible in the waveforms. Consequently, the PQRTS waveform was observed at 10 s. The ECG signal response from NSP/POE-imide/AgNPs nanohybrid electrodes can be seen in Figure 5. In comparison with conventional Ag/AgCl electrodes, fiberglass displayed remarkable flexibility, exhibiting reliability for use in fiber applications. By monitoring the electron-donor and electron-acceptor molecules in AgNPs, an ECG signal response was measured. It was shown that films with electrode sheet resistances of 8.24 × 10^−4^ Ω/sq and annealed at 300 °C displayed stability signals and had a visible P-wave, QRS-complex, and T-wave. ECGs were conducted before and after exercising. A video showing the high sensitivity of the ECG electrodes using the NSP/POE-imide/AgNPs nanohybrid is provided in the Supplementary Material (Video S1).

## 4. Conclusions

Highly sensitive ECG electrodes from electrically conductive films containing NSP/POE-imide/AgNPs nanohybrids were fabricated. The use of POE-imide as an organic dispersant and NSP as an inorganic dispersant allowed AgNPs to be well-dispersed in a DMF/water co-solvent system. The organic dispersant poly(oxyethylene) imide (POE-imide) was synthesized using poly(oxyethylene)-diamine and 4,4’-oxydiphthalic anhydride by amidation and continuous imidation. High aspect ratio polydisperse NSP (width: 100 nm; thickness: 1 nm) was prepared by the exfoliation of sodium montmorillonite (Na^+^-MMT) clay via an ion-exchange reaction with polyamine. The inorganic nanoplatelets possessed ionic charges in the form of ≡SiO^−^Na^+^ at 115 meq/100 g and were effective in stabilizing AgNPs formed by the in situ chemical reduction of silver nitrate (AgNO_3_). Through a facile coating and sintering process, hybrid polymer-mediated films could be made that exhibited low surface resistances of up to 10^−4^ Ω/sq. Furthermore, the surface resistance of the hybrid films appeared to decrease due to the interconnected network formed by the AgNP. The results indicate that these films are promising as nano-conductors in electronic devices. Silver-based electrodes show demonstrated potential not only in biosensor ECG systems for monitoring cardiac activity but also functional biological applications. However, exploring the large-scale production of these films is dependent on commercial viability and uptake. The use of dry electrodes for ECG devices is an exciting application for wearable technology featuring nanohybrid films.

## Figures and Tables

**Table 1:** Summary of sheet resistances of the NSP/POE-imide/AgNP nanohybrid films.

Sample	Weight Fraction (w/w/w)	AgNP ^a^ (wt%)	Sheet Resistance (Ω/sq) ^b^		
			160 °C	250 °C	300 °C
NSP	--	--	--	--	1.94 × 10^6^
POE-imide/AgNO_3_	1:1	--	--	1.45 × 10^−1^	2.68 × 10^−2^
NSP/POE-imide/AgNO_3_	1:10:10	1	8.27 × 10^1^	6.26 × 10^−2^	5.27 × 10^−2^
	1:20:20	1	2.74 × 10^6^	2.34 × 10^3^	5.32 × 10^−2^
	1:10:20	1	--	4.45 × 10^−2^	5.56 × 10^−2^
	1:10:35	5	2.76 × 10^3^	4.12 × 10^−4^	8.24 × 10^−4^

^a^ AgNP content in suspension. ^b^ The suspension was coated on a polyimide substrate and the sheet resistance was measured using a four-point probe.

## Supplementary Materials

The following are available online at https://www.mdpi.com/2079-4991/10/1/65/s1, Video S1: A video showing a demonstration of the LED bulbs illuminated using the highly electrically conductive films and a video depicting a highly sensitive electrocardiogram (ECG) measurement by highly electrically conductive films as flexible electrodes under dynamic activity are provided.
